# The National Dental Association

**Published:** 1914-06

**Authors:** 


### The National Dental Association.

We take special pleasure in noting the meeting of this organization, the analogue of the American Medical Association, in Rochester, July 7 to 10. The close affiliation of Dentistry with Medicine and both the professional and the practical need of making this bond still more close, are expressed by the invitation to several prominent physicians to address the meeting. It is significant that the subjects chosen are of a highly practical nature; they typify the need of joint professional discussion of common points of scientific observation and therapeutics. The medical profession is cordially invited to attend the meetings and we trust that physicians will be largely represented at Rochester.

This may be an appropriate occasion to remind both professions that they are not more widely separated, even in regard to matters of technical interest, than are the various specialties of the medical profession. It is a matter of convenience that Dentistry is organized as a separate profession, with a special degree conferred by separate schools. In professional polity, even in scientific foundation, the two professions are virtually one.

It may be well claimed that inventive genius and mechanical skill have reached their highest development in dentistry. It may also be claimed that in rapidity of development of professional spirit, of scientific discoveries and their adaptation to practical therapeutic ends, and of that very important item in any business or profession, the accomplishment of satisfactory practical results, dentistry has surpassed every other vocation, with the possible exception of electric engineering and certain limited fields in other professions. And we take pride in the fact that dentistry is, historically, a science and a profession that belongs especially to the United States of America. The very frank and general admission of this fact in Europe has undoubtedly reflected prestige on the medical profession itself.

